# Epidemic of Wheat Stripe Rust Detected by Hyperspectral Remote Sensing and Its Potential Correlation with Soil Nitrogen during Latent Period

**DOI:** 10.3390/life12091377

**Published:** 2022-09-05

**Authors:** Jing Chen, Ainisai Saimi, Minghao Zhang, Qi Liu, Zhanhong Ma

**Affiliations:** 1 Department of Plant Pathology, College of Agronomy, Xinjiang Agricultural University, Urumqi 830052, China; 2 Department of Plant Pathology, College of Plant Protection, China Agricultural University, Beijing 100193, China

**Keywords:** climate change, wheat stripe rust, hyperspectral remote sensing, identification model, soil nitrogen

## Abstract

Climate change affects crops development, pathogens survival rates and pathogenicity, leading to more severe disease epidemics. There are few reports on early, simple, large-scale quantitative detection technology for wheat diseases against climate change. A new technique for detecting wheat stripe rust (WSR) during the latent period based on hyperspectral technology is proposed. Canopy hyperspectral data of WSR was obtained; meanwhile, duplex PCR was used to measure the content of *Puccinia striiformis* f.sp. *tritici* (*Pst*) in the same canopy section. The content of *Pst* corresponded to its spectrum as the classification label of the model, which is established by discriminant partial least squares (DPLS) and support vector machine (SVM) algorithm. In the spectral region of 325–1075 nm, the model’s average recognition accuracy was between 75% and 80%. In the sub-band of 325–1075 nm, the average recognition accuracy of the DPLS was 80% within the 325–474 nm. The average recognition accuracy of the SVM was 83% within the 475–624 nm. Correlation analysis showed that the disease index of WSR was positively correlated with soil nitrogen nutrition, indicating that the soil nitrogen nutrition would affect the severity of WSR during the latent period.

## 1. Introduction

The impact of climate change on agriculture is multi-level and multi-scale. It can affect temperatures, precipitation, climate extremes, atmospheric CO_2_, crop yield, products nutritional quality and plant pests and diseases [1]. For example, climate warming not only shortens the growth period of crops and reduces the yield of corn and wheat, it also helps to improve the survival rate, fecundity, and pathogenicity of pathogens [2]. Plant diseases are one of the major threats to agriculture, which impact food production and natural systems, especially under the influence of human activities (agronomic practices, plant material movement) and climate change [3]. Plant diseases are the result of interaction between pathogens, host plants and the environment; this interaction is a continuous process referred to as the disease cycle. The quantification of the relationship between the disease cycle and weather for a given plant disease is the basis for plant disease prediction models that can be used to predict the timing and severity of plant disease in the future [4], that is, disease prediction models have also simulated potential impacts of climate change [5]. Therefore, climate change has a great influence on the plant disease risk.

Pathogens and host plants produce a series of interactions under environments, especially under climate change, and they will produce different response mechanisms. For example, WSR resistance gene *Yr39* is activated by both wheat developmental stages and climate changes [5]; climate warming leads to the expansion of wheat planting areas and the suitable range of rust fungi (*Pucciniales*). Spore germination and colonization depends on the temperature and humidity, the increase of winter temperature make for the spore overwintering, which is in favor of pathogen reproduction and causes the disease spread earlier and more serious yield loss in the following year [2]. At the same time, a suitable climate is more conducive to the pathogens reproduction and spread, but it is difficult to control the pathogens, especially in areas with high levels of inter-annual variability in climate [6]. In addition to climatic factors, soil nutrients are also an important factor affecting the occurrence of diseases. Some studies have pointed out that nitrogen application will aggravate the occurrence of wheat powdery mildew and wheat scab. Excessive nitrogen application leads to an increase in free amino acids, amides and soluble sugars, a decrease in total phenols, flavonoids, and peroxidase activities in plants, and affects the epidermal structure and metabolic activity of the host leaf [7], thereby weakening crop disease resistance and aggravating the crop disease. Meanwhile, excess nitrogen nutrition provides a favorable microclimate for the invasion, development and spread of pathogens [8]. Therefore, climate warming and soil nutrients have created favorable conditions for the outbreak of wheat diseases.

Wheat stripe rust (WSR) is caused by the fungus *Puccinia striiformis* f.sp. *tritici* (*Pst*) [9] that is widely distributed in major wheat-producing regions of the world [10,11,12]. In China, WSR causes an estimated 13.88 billion kg of loss every year [13]. *Pst* is a living obligate parasite that can spread over long distances with atmospheric pathways [14]. The infection process of *Pst* is divided into a contact period, an invasion period, a latent period, and a symptom period. The latent period is an important stage during which *Pst* multiplies and spreads in the host, and it cannot be directly perceived with the naked eye. Although the wheat does not show symptoms at this time, the parasitic relationship between the pathogen and the wheat will significantly affect the cell internal structure, pigment content, and water content of the wheat [15]. After the latent period, the disease will enter the symptom period in favorable conditions, and the fungus will rapidly expand, causing serious damage for production [16]. The duration of the latent period is one of the most important indicators for evaluating the resistance of crop cultivars [17], and it is also an important parameter in the epidemics of diseases [18]. Early detection and estimation of the dynamic of WSR during the latent period under climate change, could be obtained with more time to take prevention measures and minimize losses before the disease has become widespread.

With the rapid development of information technology, remote sensing has become increasingly used in agriculture [19,20,21,22]. Different plants have different spectral features due to their unique morphology and composition. The spectral features of plants comprise comprehensive spectral information generated by their continuous interactions with environmental factors (including biological and non-biological factors) during the growth process, and hyperspectral technology can identify the changes in the characteristic spectrum to determine the corresponding stress factors [23,24]. For example, plant disease caused by the fungal pathogenes destroyed the physical structure and physiology of crops, and manifested typical symptoms, such as foliar chlorosis, wilting or necrosis, and poor growth and development. Hyperspectral technologies have a nanometer-scale spectral resolution, which can respond to the unique disease spectrum, so the hyperspectral remote sensing is gradually applied to detect plant diseases [8,25,26]. However, traditional disease investigation techniques such as manual field scouting and quantitative PCR analysis after manual sampling [27], which has higher accuracy, is labor-intensive, inefficient, expensive and subjective, making it difficult to adapt to large-scale, non-destructive, real-time predictions of disease risk [28].

The objectives of this study were: (1) detecting the field infestation of *Pst* via the wheat canopy hyperspectral during the latent period, and (2) confirming the relationship between soil nitrogen nutrition and the severity of WSR during the latent period and symptom period. The proposed method can accurately, efficiently, and timely monitor the potential spread of WSR during the latent period, and thus is of great significance for forecasting and controlling epidemics of WSR under climate change, while also providing theoretical foundation for the optimization of nitrogen fertilizer application during the epidemic of WSR.

## 2. Materials and Methods

### 2.1. Experimental Material

The wheat cultivars used were Mingxian 169, a variety highly susceptible to *Pst*, Beijing 0045, a variety moderately susceptible to *Pst*, and Nongda 195, a variety highly resistant to *Pst.* The test strains were three races of *Pst*, CYR31, CYR32, and CYR33, that were mixed in equal proportions. The gradients of *Pst* spore suspension in 2016–2017 were 2 mg/mL, 1 mg/mL, 0.5 mg/mL, 0.25 mg/mL, and 0.125 mg/mL; in 2017–2018, these were 80 mg/L, 40 mg/L, 20 mg/L, and 10 mg/L. The above materials were provided by the Plant Disease Epidemiology Laboratory of China Agricultural University.

### 2.2. Experimental Designs

The experiment was conducted at the Kaifeng Experimental Station (34.5° N, 114.2° E) of China Agricultural University during 2016–2018. The Kaifeng Experimental Station is located in Kaifeng, Henan Province, China. There was no source of foreign stripe rust; thus, the location was suitable for artificial inoculation experiments.

**Figure 1:**
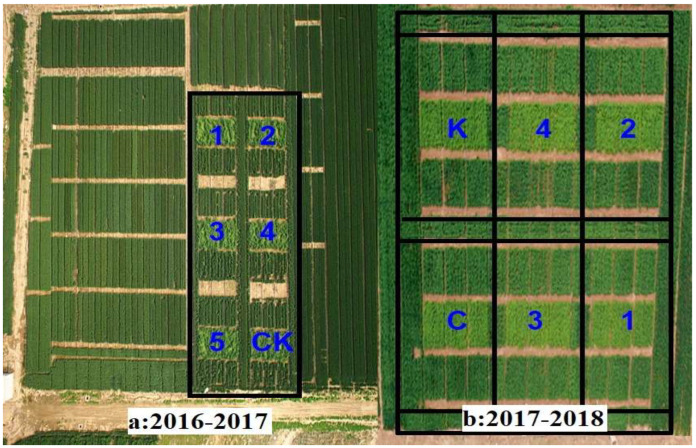
Distribution of field plots in this study. Note: Numerals, different inoculation concentrations; letters, healthy controls.

**Figure 2:**
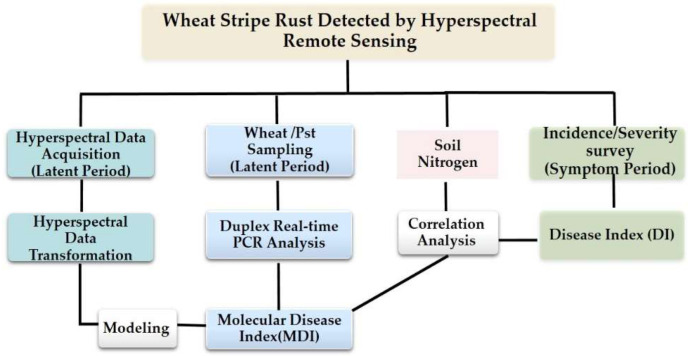
Flowchart.

A total of 54 plots (3 m × 4 m) were designed for field experiments during 2016–2017 (five inoculation concentration treatments and one healthy controls) and 2017–2018 (four inoculation concentration treatments and two healthy controls). Figure 1 showed the distribution of field plots in this study. The experiment was designed as a complete randomized block with three replicates, and there were protection rows between the plots, with an interval of 1.5 m. The sowing rate was about 225 g/plot, planted in mid-October in 2016 and 2017. Mingxian 169 was planted in the center of the community as an artificial source of induced inoculation. The field inoculation was carried out on 13 March 2017. On the 26th day after inoculation, field investigations revealed that the inoculated wheat had urediniospores, indicating that *Pst* was successfully inoculated. Therefore, the latent period of WSR in this year was 25 days. During the entire latent period, five spectroscopic measurements were performed, one day before inoculation and on the 5th, 10th, 15th, and 20th day after inoculation. The field was treated by spraying a spore suspension, carried out on20 March 2018. On the 21st day after inoculation, the presence of urediniospores was observed, and the latent period of WSR in 2018 was 20 days. During the entire latent period, five spectroscopic measurements were performed, one day before inoculation and on the 1st, 7th, 14th, and 19th day after inoculation. Meanwhile, the 0–20 cm soils of four inoculation concentration treatments with 3 replicates for each treatment were collected to analyze the correlation between the soil nitrogen and the disease index of WSR. The soil total nitrogen was determined by the semi-micro Kjeldahl method. Statistical analysis was performed using SAS v. 9.0 (SAS Institute INC., Cary, NC, USA). The framework of the study is shown in Figure 2.

### 2.3. Hyperspectral Data Acquisition and Preprocessing

An ASD spectrometer (ASD FieldSpec^®^ HandHeld™ 2, ASD Inc., Boulder, CO, USA) was used to collect wheat canopy hyperspectral data in the wavelength range of 325–1075 nm with a bandwidth of 3 nm. The wavelength accuracy was ±1 nm; the field of view was 25°, and the minimum integration time was 8.5 ms. All hyperspectral data were collected during cloudless weather between 11:30 and 14:30 (Beijing Time). The sampling height was 1.3 m.

In this study, canopy spectrum data were collected three times for each sample, and the average value was used as the canopy spectrum of the sample. During 2016–2017, a total of 4050 canopy spectra were collected in the field experiments, including 810 healthy canopy spectra before inoculation, 540 healthy canopy spectra during the same period (control), and 2700 canopy spectra of different concentration treatments during the latent period. A total of 4320 canopy spectra were obtained in 2017–2018, including 1080 canopy spectra of healthy wheat before inoculation, 1080 canopy spectra of healthy wheat during the same period (control), and 2160 canopy spectra of different concentration treatments during the latent period.

Hyperspectral reflectance is susceptible to environmental and background noise that will affect the accuracy of the signal recognition. Therefore, it was necessary to preprocess the hyperspectral data. The derivative transformation can be used to remove low-frequency background noise spectra [29]. At the same time, the logarithmic transformation of the original spectrum not only reduces the impact of changes in illumination [30] but also enhances the hyperspectral difference in the visible region [31]. In this study, the wheat canopy hyperspectral curve was processed using the ViewSpecPro software and converted into the original hyperspectral reflectance values, and the first derivative and second derivative of the hyperspectral reflectance values were calculated according to Formulas (1) and (2). There were six parameters: reflectance (R), the first derivative of R (R_1st.dv), the second derivative of R (R_2nd.dv), the logarithm of the reciprocal of R (log_10_ (1/R)), the first derivative of log10 (1/R) (log_10_(1/R)_1st.dv), and the second derivative of log_10_ (1/R) (log_10_(1/R)_2nd.dv), as the first type of hyperspectral feature. The log_10_ (1/R) is also called the pseudo absorption coefficient, as it can reflect the absorption characteristics of objects [24]. There were 22 vegetation indices, and trilateral variable parameters were used as the second type of spectral feature (refer to references [32]). At the same time, to be able to find the waveband that represented the most effective information, the 325–1075 nm wavelength range was divided into five spectral regions of the same size as the third type of spectral feature. The above three types of spectral features were modeled according to different ratios of the training set to the testing set. The hyperspectral data were statistically analyzed by ViewSpecPro (ASD), SAS 9.0, and Excel 2003.

(1) $$R^{’}\left(\lambda_{i}\right)=\frac{dR\left(\lambda_{i}\right)}{d\lambda }=\frac{R\left(\lambda_{i+1}\right)-R\left(\lambda_{i-1}\right)}{2\Delta \lambda },$$

(2) $$R^{”}\left(\lambda_{i}\right)=\frac{d^{2}R\left(\lambda_{i}\right)}{d\lambda^{2}}=\frac{R^{’}\left(\lambda_{i+1}\right)-R^{’}\left(\lambda_{i-1}\right)}{2\Delta \lambda }=\frac{R\left(\lambda_{i+2}\right)-2R\left(\lambda_{i}\right)+R\left(\lambda_{i-2}\right)}{4{\left(\Delta \lambda \right)}^{2}},$$

### 2.4. Pst Detection by Duplex Real-Time PCR during Latent Period

DNA of wheat leaves was extracted from 30 leaves per sampling point according to [33], and the DNA of *Pst* was processed as described in [34].

The primers and probes of *Pst* and wheat are refer to references [35,36]. The 20 µL reaction system of duplex PCR comprised 2.0 µL DNA template (500 pg), 2.0 µL Buffer (Mg^2+^ Free), 4.0 µL MgCl_2_ (25 µM), 2.0 µL dNTP (2500 µM), four primers of *Pst* and wheat 0.4 µL (10 µM) each, two probes 0.3 µL (10 µM) each, 0.4 µL (5 U/µL) Tag enzyme, and 7.4 µL ddH_2_O. The reaction conditions were 94 °C for 3 min pre-denaturation; 94 °C for 20 s, 57 °C for 30 s, 72 °C for 20 s, 40 reaction cycles, and fluorescence signal collection at the end of each cycle. The fluorescence intensity thresholds used for threshold cycle (CT) value collection were all set to 100. The equipment used was a MyiQ^TM^2 instrument (Bio-Rad, Hercules, CA, USA). The DNA concentration of *Pst* was calculated according to Equation (3):

(3) $$y=-0.2573x+5.4837\left(R^{2}=0.9739\right),P<0.01,$$

The DNA concentration of wheat was calculated according to Equation (4):

(4) $$y=-0.2863x+8.811(R^{2}=0.9696,P<0.01),$$

where *x* is the CT value; *y* is the logarithm (log_10_C) value of the DNA concentration. The minimum content detection limits of *Pst* DNA and wheat DNA were 0.4 pg and 0.5 ng, respectively [34].

The molecular disease index (MDI) of *Pst* was calculated according to Equation (5):

(5) MDI=PstDNA(pg)/WheatDNA(ng),

MDI reflects the DNA content of *Pst* in the latent period. The area under the disease progress curve (AUDPC) reflects the cumulative effect of the development of the disease within a certain period [37]. AUDPC can be obtained with Equation (6).

(6) $$\mathrm{AUDPC}=\sum_{i=1}^{n-1}\left(\frac{Y_{i}+Y_{i+1}}{2}\right)\left(t_{i+1}-t_{i}\right),$$

where *Y_i_* represents the MDI after inoculation, and *t_i_* represents the inoculation time.

### 2.5. Field Disease Index Acquisition

After the symptoms of wheat leaves appeared, the five-point sampling method was used to investigate the field diseases, and 30 plants were marked with GPS. For each plant, we surveyed the antepenult leaves, penultimate leaves, and flag leaves for a total of 90 leaves per point. The investigation was performed every seven days until the wheat was mature.

The incidence (*I*) and severity (*S*) of disease in the symptomatic period were recorded. Incidence is an indicator reflecting the epidemic degree of a disease and was quantified using Equation (7):

(7) $$I=\frac{n}{N}\times 100,$$

where *I* is the incidence; *n* is the number of diseased leaves, and *N* is the total number of leaves investigated. Severity (*S*) refers to the degree of damage to plants in the field, and is described in [33]. The severity of WSR was measured every seven days; the average severity was calculated according to Equation (8):

(8) $$\bar{S}=\frac{\sum \left(S\times n_{i}\right)}{n}\times 100,$$

where $\bar{S}$ is the average severity; *S* is the severity; *n_i_* is the number of diseased leaves corresponding to the severity of the disease; and *n* is the total number of diseased leaves. Disease Index (DI) is a comprehensive index that considers the incidence and severity given by Equation (9):

(9) $$DI=I\times \bar{S}\times 100,$$

where *DI* is the disease index; *I* is the incidence; and $\bar{S}$ is the average severity. AUDPC is quantified using Equation (6). SAS 9.0 software was used to analyze the correlation between MDI-AUDPC and DI-AUDPC to verify whether the MDI during the latent period of WSR could predict the actual disease’s occurrence during the symptoms period.

### 2.6. Recognition Model

On the sampling points marked by GPS, the *Pst* DNA content and the canopy hyperspectral data were obtained at the same time, and the canopy hyperspectral data were matched with MDI point-to-point. The MDI was converted into a classification label of the model. The hyperspectral data were randomly divided into a training set and a testing set. The ratios of the training set to testing set were equal to 1:1, 2:1, 3:1, 4:1, or 5:1 to compare the influence of different ratios on modeling. The DPLS and SVM methods were used to classify healthy and diseased wheat using the three types of spectral features listed above. DPLS is effective in processing data with a small sample size, high dimensionality, and multicollinearity [38] due to its dimension reduction effect [39]. Therefore, the amount of calculation can be reduced, and the calculation efficiency can be improved. SVM can better solve the problems of small samples, over-learning, nonlinearity, high dimensionality, and local minima [40]. These two recognition models were constructed based on MATLAB v.8.2 (R2013b) software (Mathworks, Natick, MA, USA). The model performance was evaluated using the overall identification accuracy.

## 3. Results

### 3.1. Wheat Canopy Spectra

**Figure 3:**
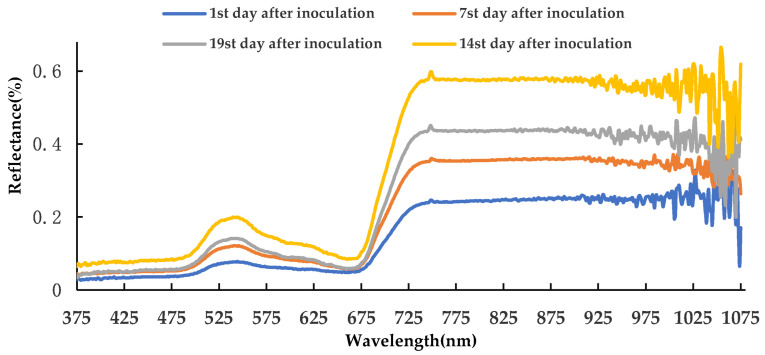
Wheat leaf spectra curves of four sampling times during latent period.

During the latent period, after averaging the canopy spectral data of the four sampling times, the spectral curve is shown in Figure 3. The variation trends of the wheat canopy spectral curves at the four sampling times were similar, and there were large differences in the range of 720–1075 nm. In the first 14 days of the latent period, the reflectance increased with the time increase, and reached the maximum on the 14th day; the reflectance values on the 19th day were lower than the 14th day. This phenomenon may be due to the rapid accumulation of *Pst*, breaking through the leaf epidermis to release spores, which changes the physiological structure and biochemical components of wheat leaves, thereby affecting the spectral reflectance. It showed that hyperspectral technology can effectively detect the latent period of WSR.

### 3.2. Correlation between MDI and DI

This study used MDI-AUDPC and DI-AUDPC for correlation analysis. The results are shown in Table 1. There was a significant correlation between MDI-AUDPC and the DI-AUDPC in 2016–2018. This indicated that the MDI of *Pst* in the latent period could predict the DI symptoms period of WSR.

### 3.3. WSR Recognition with Hyperspectral Features in the 325–1075 nm Waveband

**Figure 4:**
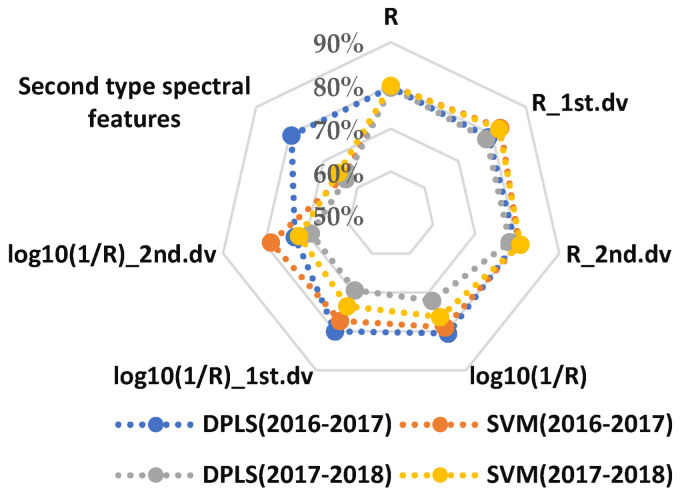
Prediction average accuracy of models resulting from different spectral features and different sampling ratios of the training set to testing set based on DPLS and SVM in all wavebands during 2016–2018.

**Figure 5:**
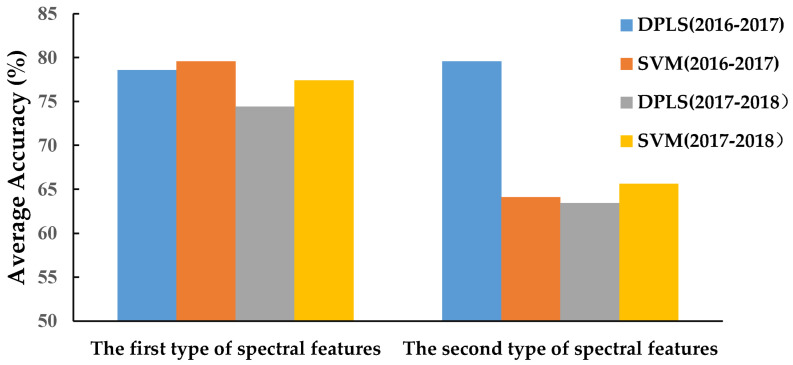
Prediction accuracy of models resulting from different spectral features and modeling methods in the 325–1075 nm waveband.

The disease recognition results during 2016–2018 are shown in Figure 4 and Figure 5. The average recognition accuracy values of the models built using DPLS in 2016–2017 and 2017–2018 were 78.56% and 74.42%, respectively. The average recognition accuracy values of the models built using SVM in 2016–2017 and 2017–2018 were 79.58% and 77.39%, respectively. The accuracy of the model built by the SVM was superior to that built using DPLS. The average accuracy of the first type of spectral feature and the second type of spectral feature in 2016–2018 was 77.49% and 68.17%, respectively. Therefore, the average accuracy and the stability of the first type of spectral feature were better than the second type of spectral feature.

Table 2 and Table 3 show that the recognition accuracy of the best models using DPLS and SVM methods were both in the range of 80–85%. The results demonstrated that it was feasible to use the wheat canopy hyperspectral data and the MDI of *Pst* to establish a mathematical model to detect the occurrence of WSR.

### 3.4. WSR Recognition with Hyperspectral Features in the Sub-Waveband Range

#### 3.4.1. Recognition Results of the DPLS Model in 2016–2017

**Figure 6:**
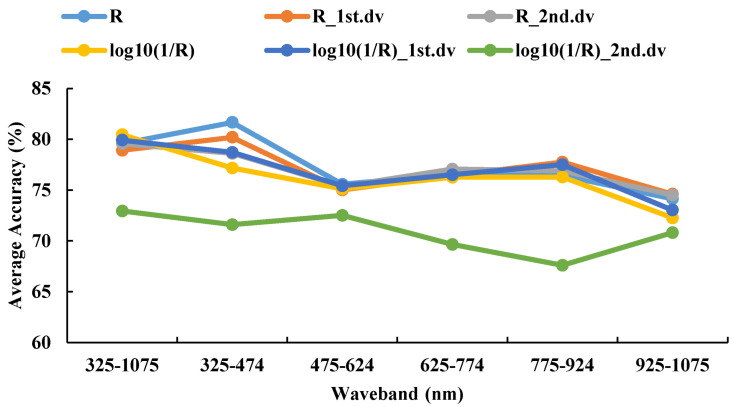
The testing sets average accuracy based on different spectral features and the same waveband using DPLS in 2016–2017.

**Figure 7:**
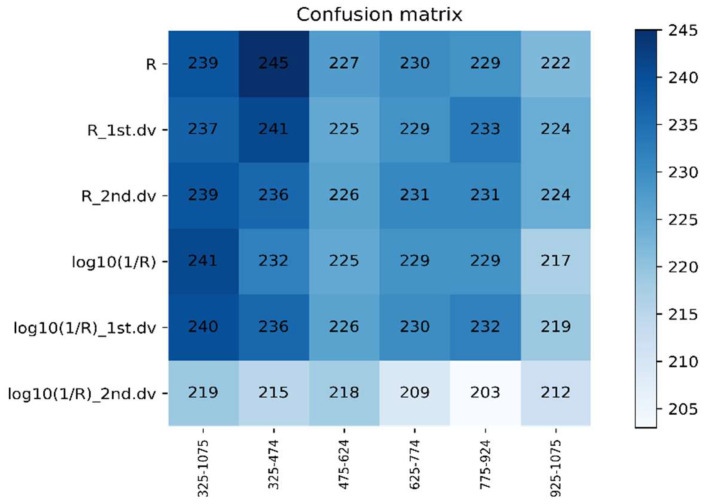
The confusion matrix based on different spectral features and the same waveband using DPLS in 2016–2017.

The 325–1075 nm waveband was divided into five spectral regions (325–474 nm, 475–624 nm, 625–774 nm, 775–924 nm, and 925–1075 nm) of the same size. The DPLS algorithm was used to identify WSR based on different ratios of the training set to testing set and different spectral features during 2016–2017. It can be seen in Figure 6 and Figure 7 that the accuracy of the DPLS model built by the pseudo absorption coefficient was relatively good in all wavebands, and the average accuracy of the testing set was 80.45%.

The recognition accuracy of the DPLS model had the highest values at the waveband range 325–474 nm when using R as the spectral feature, where the average accuracy of the testing set was 81.66%. These values were given priority as the candidate waveband and spectral feature for model establishment. The best model was based on 325–474 nm with R as the spectral feature, with a sampling ratio of 4:1. The accuracy of the testing set was 85.19%.

#### 3.4.2. Recognition Results of SVM Model in 2016–2017

**Figure 8:**
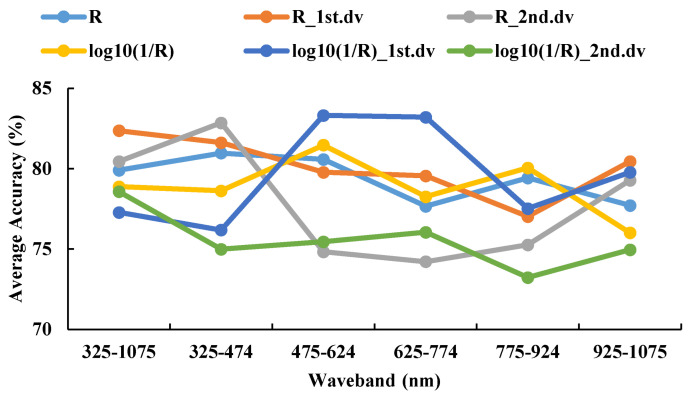
The testing sets average accuracy based on different spectral features and the same waveband using SVM in 2016–2017.

**Figure 9:**
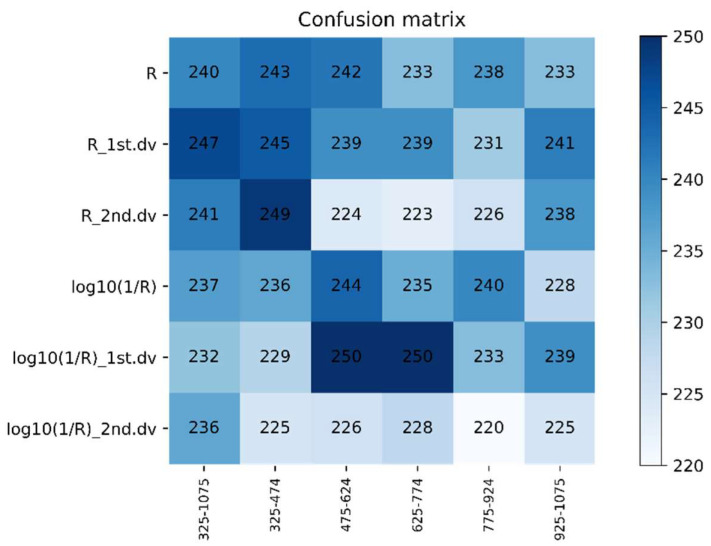
The confusion matrix based on different spectral features and the same waveband using SVM in 2016–2017.

When the R_1st.dv was used as the spectral feature in all wavebands, the average accuracy of the testing set was 82.37%. When log_10_(1/R)_1st.dv was used as the spectral feature, the model showed a peak within the 475–624 nm range, and the average accuracy of the testing set was 83.32% (Figure 8 and Figure 9). These values were used as the preferred waveband and spectral feature for the model establishment. The best model was characterized by the R_2nd.dv in the range of 325–474 nm. When the sampling ratio was 4:1, the optimal parameters of the model were c = 588.1336; g = 1024, and the accuracy of the testing set was 87.04%.

#### 3.4.3. Recognition Results of DPLS Model in 2017–2018

**Figure 10:**
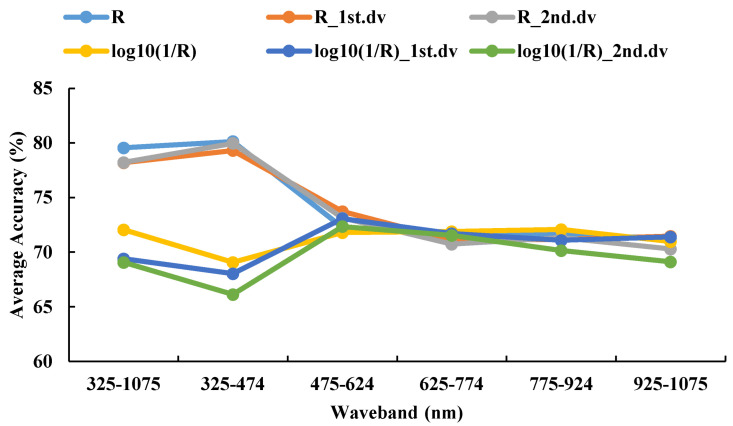
The testing set average accuracy based on different spectral features and the same waveband using DPLS in 2017–2018.

**Figure 11:**
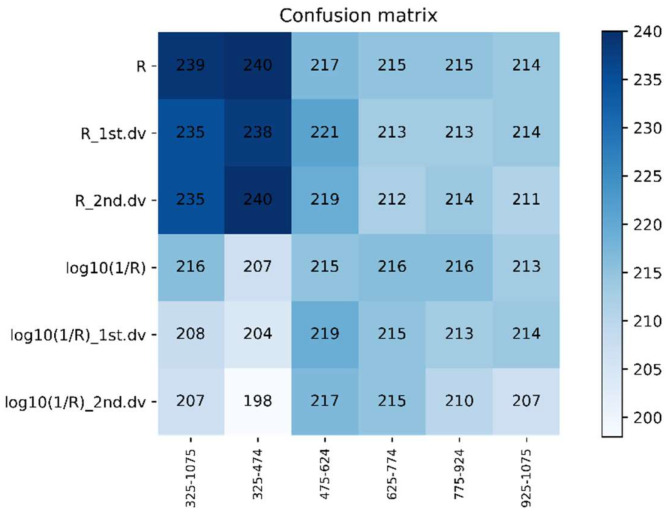
The confusion matrix based on different spectral features and the same waveband using DPLS in 2017–2018.

The recognition accuracy of the model built by R was relatively good in all wavebands, and the average accuracy of the testing set was 79.57%. When the waveband range was 325–474 nm and R was the spectral feature, the average accuracy of the model was the best at 80.14% (Figure 10 and Figure 11), and these values can be given priority as the candidate waveband and spectral feature for model establishment. The best model comprised 325–474 nm as the range; the original spectrum was the spectral feature; the sampling ratio was 4:1, the accuracy of the testing set was 81.60%.

#### 3.4.4. Recognition Results of SVM Model in 2017–2018

**Figure 12:**
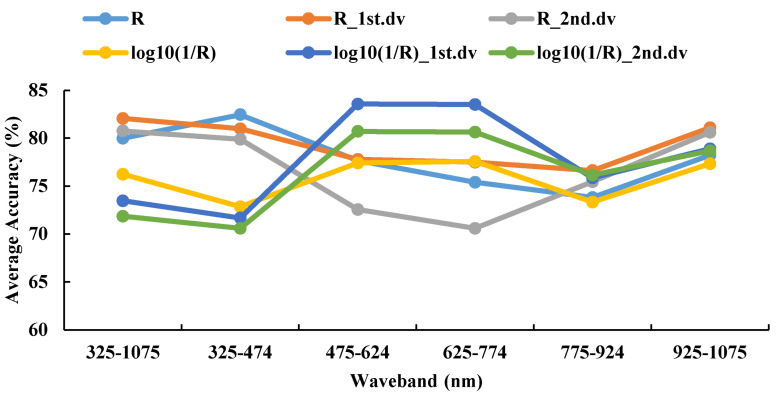
The testing set’s average accuracy based on different spectral features and the same waveband using SVM in 2017–2018.

**Figure 13:**
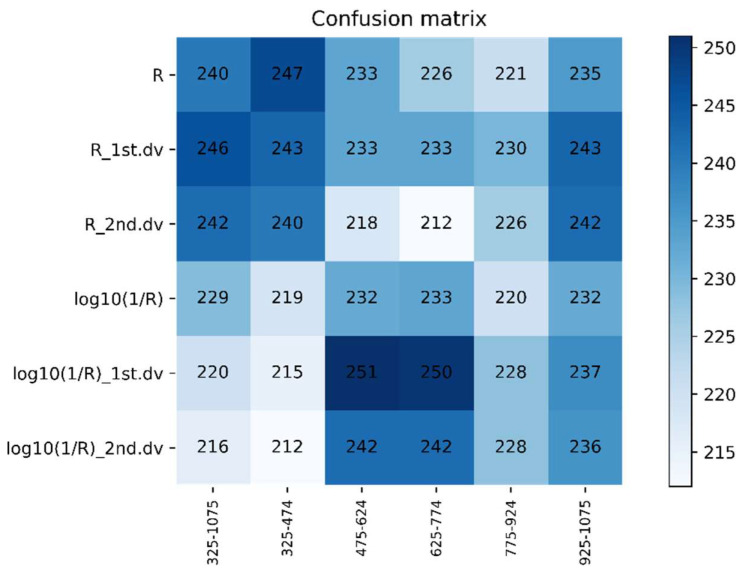
The confusion matrix based on different spectral features and the same waveband using SVM in 2017–2018.

When the R_1st.dv was used as the spectral feature, the recognition accuracy was the best in all wavebands, and the average accuracy of the testing set was 82.05%. When the first derivative of the absorption coefficient was used as the spectral feature within the range 475–624 nm, the average accuracy of the model was the highest at 83.56% (Figure 12 and Figure 13), and thus these values could be prioritized as the candidate waveband and preferred spectral feature for model establishment. The best model used the 325–474 nm range, the original spectrum as the feature, and a sampling ratio of 4:1; the optimal parameter c was 2.2974; g was 337.7940, and the accuracy of the model on the testing set was 85.76%.

### 3.5. Correlation between Soil Nitrogen Nutrition and WSR Severity

The correlation between the severity of WSR and soil nitrogen nutrition of different inoculation concentrations of pathogens and different varieties of wheat during the latent period and symptom period were analyzed (Table 4). The results showed that the severity of WSR was positively correlated with soil nitrogen nutrition under artificial inoculation conditions. The correlation between different inoculation concentrations and soil nitrogen nutrition was extremely significant. The correlation between the symptom period and soil nitrogen was slightly higher than the latent period. As the inoculation concentration increased, the severity of the disease gradually increased. With the decrease of inoculation concentration, the correlation between disease index and soil nitrogen nutrition gradually decreased. The disease severity of different wheat resistant varieties under artificial inoculation conditions showed the same regularity as the natural incidence. The susceptible varieties (Mingxian169) had the higher correlation coefficient. Middle-resistant varieties and resistant varieties showed a weaker correlation. Correlation analysis showed that soil nitrogen nutrition was correlated with the occurrence of WSR during the latent and symptom period, but the inoculation concentration and variety had a greater impact on it.

## 4. Discussion

### 4.1. Recognition of Wheat Stripe Rust with Hyperspectral Remote Sensing

Based on the results, it appears feasible to establish models based on the DPLS and SVM methods to identify WSR during the latent period in field settings. Meanwhile, the spectral ranges and spectral characters were good candidates for the fingerprint method for the rapid estimation of *Pst*-infected wheat leaves.

The two-year test results showed that, based on the R spectral feature, the average recognition accuracy of the DPLS algorithm was about 80% in the bands of 325–474 nm. Based on the first derivative of the absorption coefficient as the spectral feature within the 475–624 nm range, the average recognition accuracy of the SVM algorithm was about 83%. In other words, the visible part of the spectrum (400–680 nm) was relevant in distinguishing between the latent period and the symptomatic period of WSR, suggesting that *Pst* infection alters the spectral properties of wheat leaves. For fresh plants, leaf reflectance in the visible spectrum is usually low due to the absorption of pigments (e.g., chlorophyll and anthocyanin) in leaves. However, in the latent period of *Pst* infected wheat leaves, although the external morphology has not changed, internal changes have occurred that destroy leaf pigments, leading to changes in the color of plant leaves and increases in spectral reflectance [41]. Each host–pathogen interaction is unique, and the resulting spectral changes are also unique. By monitoring these spectral changes it is possible to analyze the severity and spread of diseases. This analysis has further confirmed that the canopy spectrum of WSR has a very significant correlation with the disease index.

The highest recognition accuracy rate was 87.04% in 2016–2017, and the highest rate was 85.76% in 2017–2018. The recognition accuracy of the model built in the field experiments was generally lower than for the indoor experiment model [42]. The reason may be due to the complex field environment, the many interference factors, such as illumination intensity, and soil nutrients. Therefore, continuing to optimize the model parameters, wavebands, and spectral characters can improve the model’s recognition accuracy. Meanwhile, the detection limit of *Pst* by measuring the hyperspectral data of the wheat canopy with a spectrometer will be the focus of our further studies.

### 4.2. Correlation between Soil Nitrogen Nutrition and WSR Severity

Climatic factors are the direct factors leading to the WSR, because of its airborne. Additionally, a warmer climate will lead to more days for sporulation, shorter latent period and higher spore reproductive rate will lead to more spores produced in the suitable temperatures [2]. Therefore, it is particularly important to be able to perform a high-throughput screening WSR during the latent period. However, there are many factors affecting the severity of the disease, such as varieties, fertilization, and soil environment. When the crop reaches the optimal “nutrient balance” for growth, the disease resistance is the strongest, but it will change as the nutrient status deviates from the optimal growth degree [43]. Reasonable fertilization promotes the balance of various nutrients in wheat, which is conducive to the control of WSR. It has been reported that high nitrogen application results in increased wheat rust severity [2], and wheat rust disease and N deficiency both cause changes in foliar pigments that result in chlorosis [44]. Therefore, it is crucial for hyperspectral remote sensing to distinguish disease infection from nitrogen nutrient effects during the latent period. We will continue to verify the influence of different nitrogen application levels on disease severity, spectral diagnosis, and the effects of WSR and soil nitrogen interaction on spectral characteristics of wheat during the latent period.

## 5. Conclusions

In this study, we developed a high degree of accuracy approach for high-throughput detection WSR directly during the latent period.

1. In the 325–1075 nm waveband, the average recognition accuracy of the model built by SVM was better than that using DPLS. The average accuracy of the model built by the first type of spectral feature was better than the model using the second type of spectral feature. The average accuracy values of the DPLS and SVM methods were 75–80%, and the accuracy of the best-performing model was between 80–85%.
2. In the sub-wavebands, the models built based on the DPLS method with the best accuracy in two years were all concentrated in the 325–474 nm range using the original spectrum (R) as the spectral character. The models built based on the SVM method with the best recognition accuracy in the two years were concentrated in the 475–624 nm range using the first derivative of the pseudo absorption coefficient (log_10_(1/R)_1st.dv) as the spectral feature.
3. There was a significant positive correlation between wheat stripe rust and soil nitrogen nutrients during latent period and symptom period, which also provided the theoretical basis for more accurate remote sensing monitoring on the wheat stripe rust.

## Figures and Tables

**Table 1:** Correlation analysis between MDI-AUDPC and DI –AUDPC in different years.

Year	Correlation Coefficient	Significance Level	Regression Equation	R^2^	Root Mean Square Error
2016–2017	0.84840	<0.0001	y = 0.0415 + 11.973X	0.7198	0.1221
2017–2018	0.90056	<0.0001	y = 0.6176 + 6.4193X	0.8110	3.1608

**Table 2:** Prediction accuracy of the best models based on DPLS in the 325–1075 nm waveband.

Year	Spectral Features	The Ratio of the Training Set to Testing Set	The Principal Component Number	Accuracy	F1 Score	Matthews Correlation Coefficient
2016–2017	log_10_(1/R)	4: 1 4: 1	30	84.57	84.21	82.75
2017–2018	R		30	82.29	81.82	80.84

**Table 3:** Prediction accuracy of the best models based on SVM in the 325–1075-nm waveband.

Year	Spectral Features	The Ratio of the Training Set to Testing Set	Optimal Parameter		Accuracy	F1 Score	Matthews Correlation Coefficient
			Best c	Best g			
2016–2017	R_1st.dv	3: 1	6.9644	64	83.17	83.15	82.23
2017–2018	R_1st.dv	4: 1	2.2974	64	84.03	83.65	82.19

**Table 4:** Correlation between soil total nitrogen nutrition and disease index of WSR.

Growth Stage	Inoculation Concentration (mg/L)	Mingxian169 Disease Index	Beijing0045 Disease Index	Nongda195 Disease Index
Latent	80	0.916 **	0.574	0.517
Period	40	0.922 **	0.493	0.513
	20	0.801 *	0.354	0.487
	10	0.599	0.277	0.101
Symptom	80	0.982 **	0.673	0.599
Period	40	0.895 **	0.54	0.466
	20	0.838 *	0.13	0.084
	10	0.711	0.063	0.058

Note: *, ** Indicate significant differences at the 5% and 1% levels, respectively.

## Data Availability

The data sets generated during and/or analyzed during the current study are available from the corresponding author upon reasonable request.
